# High-Throughput Sequencing Reveals Hypothalamic MicroRNAs as Novel Partners Involved in Timing the Rapid Development of Chicken (*Gallus gallus*) Gonads

**DOI:** 10.1371/journal.pone.0129738

**Published:** 2015-06-10

**Authors:** Wei Han, Jianmin Zou, Kehua Wang, Yijun Su, Yunfen Zhu, Chi Song, Guohui Li, Liang Qu, Huiyong Zhang, Honglin Liu

**Affiliations:** 1 College of Animal Science & Technology, Nanjing Agricultural University, Nanjing, PR China; 2 National Chickens Genetic Resources, Poultry institute, Chinese Academy of Agricultural Science, Yangzhou, PR China; Institute of Zoology, Chinese Academy of Sciences, CHINA

## Abstract

Onset of the rapid gonad growth is a milestone in sexual development that comprises many genes and regulatory factors. The observations in model organisms and mammals including humans have shown a potential link between miRNAs and development timing. To determine whether miRNAs play roles in this process in the chicken (*Gallus gallus*), the Solexa deep sequencing was performed to analyze the profiles of miRNA expression in the hypothalamus of hens from two different pubertal stages, before onset of the rapid gonad development (BO) and after onset of the rapid gonad development (AO). 374 conserved and 46 novel miRNAs were identified as hypothalamus-expressed miRNAs in the chicken. 144 conserved miRNAs were showed to be differentially expressed (reads > 10, *P* < 0.05) during the transition from BO to AO. Five differentially expressed miRNAs were validated by real-time quantitative RT-PCR (qRT-PCR) method. 2013 putative genes were predicted as the targets of the 15 most differentially expressed miRNAs (fold-change > 4.0, *P* < 0.01). Of these genes, 7 putative circadian clock genes, *Per2*, *Bmal1/2*, *Clock*, *Cry1/2*, and *Star* were found to be targeted multiple times by the miRNAs. qRT-PCR revealed the basic transcription levels of these clock genes were much higher (*P* < 0.01) in AO than in BO. Further functional analysis suggested that these 15 miRNAs play important roles in transcriptional regulation and signal transduction pathways. The results provide new insights into miRNAs functions in timing the rapid development of chicken gonads. Considering the characteristics of miRNA functional conservation, the results will contribute to the research on puberty onset in humans.

## Introduction

Puberty is a milestone development phase that leads to the completion of sexual maturation and the achievement of reproductive capacity in mammals as well as in humans. Although precocious puberty is a pathologic status for humans, it is an economic trait in poultry breeding and production. In comparison to mammals and humans, chicken’s sexual development is very distinctive. Generally, the hens reach sexual maturity and start to produce eggs at 4~5 months of age. Lay of the first egg is regarded as the sign of sexual maturity [1]. The females post-hatching have no clear definition of puberty before sexual maturity, and no estrus behavior and cycles after sexual maturity. In early period, the gonads of females develop slowly and will enter into rapid growth about 30 days before the age at first egg, which is accompanied by higher increase rate of comb sizes. In some respects, this transition in chickens is analogous to puberty onset in mammals that is signified by quick changes of sexual organs and emergence of the secondary sex characters. Despite intense researches have been devoted to the physiological characteristics during sexual maturity [2, 3], the regulatory mechanisms that underlie onset of the rapid gonad development in the chicken is not well understood.

Onset of the rapid gonad development in the chicken, as puberty onset in the mammals is controlled by many factors and multiple regulatory pathways. In the past two decades, various metabolic signals and environmental cues have been found to play important roles in puberty onset of mammals [4, 5]. Yet, they only act as permissive signals that allow puberty to occur but do not cause puberty [6]. Now, it is clear that an increase in pulsatile gonadotrophin releasing hormone (GnRH) release from the hypothalamus is the determined event that causes puberty to occur, which functionally leads to the reactivation of hypothalamic-pituitary-gonad (HPG) axis. The coordinated modifications in transsynaptic and glial inputs to the GnRH neuronal network have been found to contribute to this change [7]. Some newly identified components in this network, such as kisspeptin [8, 9] and neurokinin B (NKB) [10], have deepened our understanding of the neuroendocrine and molecular bases for the control of puberty onset.

However, there is no doubt that no isolated pathway or component is solely responsible for the control of puberty onset. Genomic and system biological approaches have suggested a set of genes are involved in the initiation of puberty [7]. Of note, genome-wide association studies show *LIN28B* gene is associated with the age at menarche [11], breast development and adult height [12] in humans. Furthermore, mice that over-expression of *Lin28A* have delayed puberty [13]. *LIN28A* and *LIN28B* are the homologs of heterochronic gene *LIN28* in *Caenorhabditis elegans*, which encode RNA-binding proteins that inhibit the maturation of the let-7 family microRNAs (miRNAs) [14]. These observations imply potential links between miRNAs and development timing [15].

miRNAs are small non-coding RNA molecules (~22 nt in length) that control gene expression by post-transcription manner. By binding to the complementary sequences in the 3,-untranslated regions (UTRs) of target mRNAs, miRNAs degrade or inhibit the translation of mRNAs. miRNAs have been found to participate in regulation of almost all biological and physiological processes [16]. As accurate micromanagers, miRNAs are very suitable for determining the time of developmental events. In fact, lin-4 and let-7 family miRNAs are originally discovered as regulators of developmental timing in *C*.*elegans* [17, 18], which precisely control the transition of four larval stages by down-regulating particular targets [19]. Excitingly, miRNAs are also showed to perform analogous development timing functions in other species. In *Arabidopsis thaliana*, miR-156 and miR-172 define a separate endogenous flowering pathway [20]. In *Drosophila*, *bantam* miRNA promotes systemic growth by connecting insulin signaling and ecdysone production [21]. Very recently, Alvarellos *et al* (2013) [22] report that the developmental changes of *Lin28*/*let-7* expression in hypothalamus may lead to puberty onset of rats. Therefore, miRNAs may represent novel partners involved in timing the transition of developmental events. However, little is known about the regulatory mechanisms of miRNAs in timing the rapid growth of chicken gonads.

In the present study, we used Solexa sequencing method to investigate the expression profile of miRNAs in the hypothalamus of Wenchang chicken, a famous Chinese indigenous breed for its early maturity characters, during onset of the rapid gonad development. We focus on the hypothalamus tissue because it is the major site responsible for onset of the rapid gonad development. The results show that many miRNAs are differentially expressed during onset of the rapid gonad development. Target prediction and functional analysis of the differentially expressed miRNAs reveal several important pathways might be related to onset of the rapid gonad development. Together, our findings provide new insights into miRNA functions in timing the rapid development of chicken gonads. Considering the characteristics of miRNA functional conservation, the results will contribute to the puberty onset research in humans.

## Materials and Methods

### Ethics statement

The Wenchang chicken breed used in our study is not an endangered species. The animals were allowed access to feed and water ad libitum. Before sacrifice, the chickens were anaesthetized by giving them water mixed with diazepam and ethanol. When they became unconscious, the electric shocks were carried to minimize suffering. All animal experiments were approved by both the Institutional Animal Care and Use Committees in College of Animal Science&Technology, Nanjing Agricultural University, Nanjing, China, and Poultry institute, Chinese Academy of Agricultural Science, Yangzhou, China.

### Measurement of the gonad development in chickens

Wenchang chickens were from Hainan Luoniushan Wenchang chicken breeding farms. The comb size, ovary weight, oviduct length and follicle size for hens (n = 10) were measured from 5 to 17 weeks. Comb height was taken as the distance from the highest point on the second blade to the base of the skull. Width was taken across the distance from the attachment of the front blade to the base of the skull. Oviduct length was taken as the distance from the infundibulum segments to the cloaca parts. The transition timing of the rapid gonad development was judged by changes of these indexes. The phases just *pre-* and *post-* the initiation of rapid gonad development were termed as before onset of the rapid gonad development (BO) and after onset of the rapid gonad development (AO) in this study.

### Collection of hypothalamus tissues

A mating family, consisting of one cock and fifteen hens at the age of 43 weeks, was set up. The fifteen hens had similar body weights (coefficient of variance (CV) < 10%) and the same first laying day. Artificial insemination was carried out and the fertilized eggs were collected for 10 days. After hatching and sex separation, the full-/ half-sib females were fed to 13 weeks under standard feeding and management conditions. The individuals with healthy performance and body weight in 5% upper and lower limits of average values were selected (16/26, average body weight = 1355.2g), and then sacrificed, the hypothalamus tissues were removed and immediately frozen in liquid nitrogen. The comb size, ovary weight, oviduct length and follicle size of these individuals were also measured. The sampling was finished within two hours (a.m. 9:00~11:00). Sampled animals were divided into AO and BO groups according to their status of sexual development.

### Small RNA library preparation and sequencing

Dissected hypothalamus tissues from 6 chickens with similar body weights (CV < 10%) were used in this study, including 3 BO animals and 3 AO animals. On relationships, each group contained one full-sib and two half-sibs. Total RNA was extracted from the hypothalamus using mirVana Isolation Kit (Ambion, USA) according to the manufacturer’s instructions. Total RNA quality was checked with a Bioanalyzer 2100 (Agilent Technologies, USA). The RIN was > 8.0 and A260/A280 was > 2.1 for all samples. Total RNAs were stored at -80°C for further use.

Total RNA of chickens from the same status were mixed with equal amounts to construct two pooled libraries: BO and AO. The overall flow of the sequencing procedure is as follows: the small RNAs ranging from 18 to 35nt in length was purified from 15% polyacrylamide gels, then ligated to 5, and 3, adapters. Reverse transcription was performed, and followed by PCR amplification. The purified PCR products were used directly for cluster generation and sequencing analysis using the Illumina’s Solexa Sequencer according to the manufacturer’s instructions (Shanghai Biotechnology Co., Ltd, China).

### Sequence data analysis

Conserved miRNAs identification: The raw reads obtained from Solexa sequencing were filtered by deleting poor quality reads, adaptor pollution reads and reads less than 18nt in length. The clean reads of small RNAs were aligned to the reference chicken (*G*.*gallus*) genome. The sequences that correspond to known miRNAs were determined by perfect sequence matching to the miRNA database (miRBase 20.0). Other small RNAs (rRNA, tRNA, pirna and snoRNA) were annotated by blasting against the Rfram database (http://sanger.ac.uk/software/Rfam), the piRNA database (http://pirnabank.ibab.ac.in/) and the ncRNA database (http://biobases.ibch.poznan.pl/ncRNA/). All alignments were done by CLC genomics workbench 5.5.

Novel miRNAs discovery: The unannotated small RNA sequences were aligned to the reference chicken (*G*.*gallus*) genome to find precursor sequences for novel miRNAs. Novel miRNAs were predicted by miRCat tools with stem-loop structure.

Differentially expressed miRNAs analysis: The R package DEGseq software was used to identify differentially expressed miRNAs. The number of conserved miRNAs was normalized to the total number of reads in each sample that matched the chicken (*G*.*gallus*) genome. P-values for differentially expressed miRNAs were calculated using the MA-plot-based random sampling model (MARS).

The raw sequences for BO and AO had been deposited to the NIH Short Read Archive (Accession Number SRP055698).

### Quantitative real-time PCR analysis

For miRNA, total RNAs were reverse-transcribed by miScript II RT kit (QIAGEN, USA). The Oligod (T) RT primers were used. snRNA U6 was used as the control for the real-time quantitative RT-PCR (qRT-PCR). The PCR reactions were performed as follows: 95°C for 2 minutes, then 40 cycles with 94°C for 15 seconds and 60°C for 1 minute.

For mRNA, the first cDNA chain was generated by iScript cDNA synthesis kit (BIO-RAD, USA). The primers were designed by Primer Premier (V6.0) software. GAPDH was used as the control for the qRT-PCR. The PCR reactions were performed as follows: 50°C for 2 minutes, 95°C for 10 minutes, then 40 cycles with 95°C for 15 seconds and 60°C for 1 minutes.

Samples were the redundant hypothalamus tissues of the six ones that used for high-throughput sequencing, not pooled, so n = 3 for BO and AO respectively. All experiments were performed on ABI 7900 HT sequence detection system. Every reaction was carried out with 3 replicates. Relative expression levels were determined using the 2^-ΔCT^ method. Statistical significance of the expression change was determined by t-test in SPSS 20.0.

### MiRNA target prediction and functional analysis

Target genes of differentially expressed miRNAs were predicted using TargetScan and miRanada software. To generate a higher prediction accuracy, the putative target genes with context score percentile < 50 were discarded in TargetScan, and the ones with maximum energy > -10 were also deleted in miRanda. Gene Ontology (GO) annotation and Kyoto Encyclopedia of Genes and Genomes (KEGG) pathway analysis were retrieved using DAVID (http://david.abcc.ncifcrf.gov/). Only the GO terms and KEGG pathways with *P* < 0.05 were taken into account.

## Results

### Measurement of the gonad development in chickens

To divide pubertal stages in Wenchang chicken, we measured the progressive changes of gonad tissues, follicle and comb sizes from 5 to 17 weeks (Table 1). The results showed that the ovary and oviduct developed slowly and the follicles kept primitive in early period of development. This period would last about 12 weeks. Then, the gonad tissues developed explosively, the ovary weight and oviduct length of 13 weeks increased 125% and 120% respectively compared to that of 12 weeks, accompanied by emergence of pre-hierarchical follicles. A significant change of the secondary sex characteristic can be observed during this period was the skin color around eyelids becoming deep red from yellow or pale red. Subsequently, about 20~30 days later, hens could reach sexual maturation and lay the first egg. Circulating Luteinizing Hormone (LH) levels were also measured in our study, but they were not considered because of overlaps across stages and larger value variability. Based on these, we determined the crucial “timing” for transition of the rapid gonad development for hens is about 13 weeks in Wenchang chicken.

**Table 1:** Measurement of comb size and gonad tissues during different development stages.

Weeks	Diameter of the largest follicle (mm)	Ovary weight (g)	Oviduct length (cm)	Comb size (height × width, mm)
5	< 1mm	0.12±0.01	2.47±0.28	4.5×12.7
6	< 1mm	0.16±0.02	2.52±0.30	6.1×15.0
7	< 1mm	0.22±0.02	3.03±0.42	8.2×16.1
8	< 1mm	0.30±0.02	3.46±0.38	9.6×18.1
9	< 1mm	0.41±0.03	4.30±0.37	10.5×22.9
10	< 1mm	0.46±0.05	5.27±0.55	13.0×25.4
11	< 1mm	0.53±0.04	6.07±0.73	16.8×28.6
12	< 1mm	0.60±0.10	7.16±0.74	20.3×30.3
**13**	**3.24±0.55**	**1.35±0.11**	**15.68±1.83**	**25.4×35.2**
14	7.03±0.86	2.26±0.29	17.85±1.97	28.1×37.0
15	12.57±1.87	10.5±1.24	22.40±3.06	29.0×37.2
16	20.60±1.35	18.4±2.16	27.67±2.35	29.9×37.5
17	26.14±1.03	40.8±3.53	43.57±3.80	30.7×39.4

For each phase n = 10.

### Small RNA library construction and sequencing

To identify potential miRNAs that contributed to onset of the rapid gonad development in chickens, we constructed two small RNA libraries from hypothalamus collected from animals in two different developmental status: before onset of the rapid gonad development (BO) and after onset of the rapid gonad development (AO). Six hens, two full-sibs and four half-sibs with similar body weights (CV < 10%) were selected at the age of 13 weeks. Each library consisted of one full-sib and two half-sibs. The 3 BO animals were judged by the criteria: ovary weight 0.5g ~ 0.6g, oviduct length 6.0cm ~ 7.0cm, and the largest follicle size < 1mm, which meant the ovary and oviduct still keep slow development. Whereas the 3 AO animals were judged by the criteria: ovary weight 1.5g ~ 2.0g, emergence of pre-hierarchical follicles, the largest follicle size 3.0mm ~ 5.0mm, and the oviduct length 15.0cm ~ 17.0cm, which meant the gonad tissues had just entered into rapid development.

**Fig 1:**
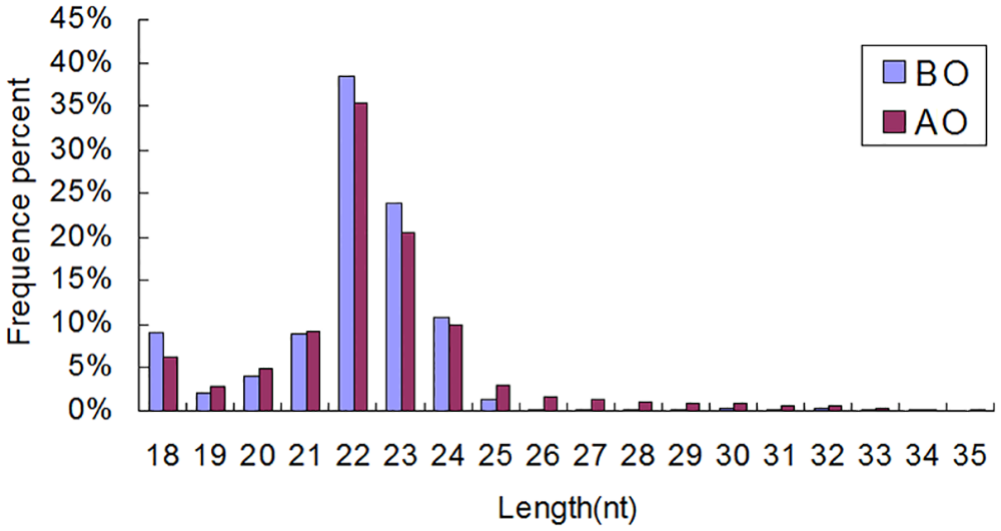
Length distribution of the clean reads in BO and AO. The X axis indicates sequence size from 18nt to 35nt. The Y axis indicates the percents of reads for every given size. The majority of sequences for both libraries were 21nt ~ 24nt. BO, before onset of the rapid gonad development; AO, after onset of the rapid gonad development.

High-throughput Solexa sequencing of these two small RNA libraries yielded 14,668,149 (BO) and 10,100,202 (AO) raw reads. After filtered low quality sequences, 10,678,413 (72.8%) and 8,160,964 (80.8%) clean reads were obtained for BO and AO respectively. The histograms of the reads length distribution showed similar trends between two libraries, and the majority of sequences were 21nt ~ 24nt in length (Fig 1). Of these, 8,110,062 (BO) and 7,414,538 (AO) reads were annotated by mapping to established small RNA databases (Table 2). The high percentage of miRNAs, 82.5% for AO and 65.1% for BO, indicated that miRNAs may represent the majority of small RNAs involved in puberty onset. 843,432 and 173,400 unique small RNAs were revealed, and 112,074 (BO) and 51,887 (AO)small RNAs were annotated.

**Table 2:** Mapping statics of Solexa sequencing reads.

Matches	Reads	Percent (%)	Unique sRNAs	Percent (%)
	(BO vs AO)	(BO vs AO)	(BO vs AO)	(BO vs AO)
	10678413/8160964	100%/100%	843432/173400	100%/100%
Unannotated	2568351/746426	24.1%/9.1%	731358/121513	86.7%/70.1%
Annotated	8110062/7414538	75.9%/90.9%	112074/51887	13.3%/29.9%
With miRBase	5282660/6120130	65.1%/82.5%	17572/17395	15.7%/33.5%
With ncrna	607692/373011	7.5%/5.0%	30597/15357	27.3%/29.6%
With pirna	351808/33152	4.3%/0.4%	7686/1788	6.9%/3.4%
With Rfam	1867902/888245	23.0%/12.0%	56219/17347	50.2%/33.4%

BO indicates before onset of the rapid gonad development, AO indicates after onset of the rapid gonad development.

### Identification of conserved miRNAs

To identify conserved miRNAs in chicken hypothalamus, we aligned the small RNAs to current miRBase (Release V20.0). Sequences with perfect matching to known chicken (*G*.*gallus*) miRNAs were considered as conserved miRNAs. In total, 331 and 323 conserved sequences were annotated for BO and AO respectively, as chicken miRNAs. Among them, 280 were sorted as common miRNAs, 51 as BO-specific miRNAs and 43 as AO-specific miRNAs. The conserved miRNAs expressed in the chicken hypothalamus were listed in S1 Table.

### Discovery of novel miRNA candidates

To predict novel miRNAs, we analyzed the 2,568,351 (BO) and 746,426 (AO) unannotated reads using miRCat tools. The prediction strategy was as follows: (1) the sRNAs matched to chicken genome; (2) hairpin miRNAs could fold into secondary structures and mature miRNAs were present in one arm of the hairpin precursors, with the Rnadfold p-value lower than 0.05; (3) the secondary structures of the hairpins were steady, with the adjusted minimum free energy < -25 kcal/mol. A total of 46 novel miRNAs conformed to these criteria. Novel miRNAs expressed in the chicken hypothalamus were listed in S2 Table. The abundance of these novel miRNAs was relatively lower. So, this could explain why they were not found in previous studies.

### Differential expression profiles of conserved miRNAs between BO and AO

To compare the differential expression of miRNAs in the hypothalamus of BO versus AO chickens, the number of miRNAs in each sample was normalized to the total number of reads (BO/AO = 1.15732). In total, 162 miRNAs were considered to be differentially expressed (*P* < 0.05). To obtain a reliable list, we restricted our analysis to only those miRNAs with read counts > 10 in at least one sample. This resulted in 144 differentially expressed miRNAs, with 53 up-regulated and 91 down-regulated miRNAs between BO and AO. The differentially expressed miRNAs between BO and AO were listed in S3 Table.

### Validation of differential expression miRNAs

**Fig 2:**
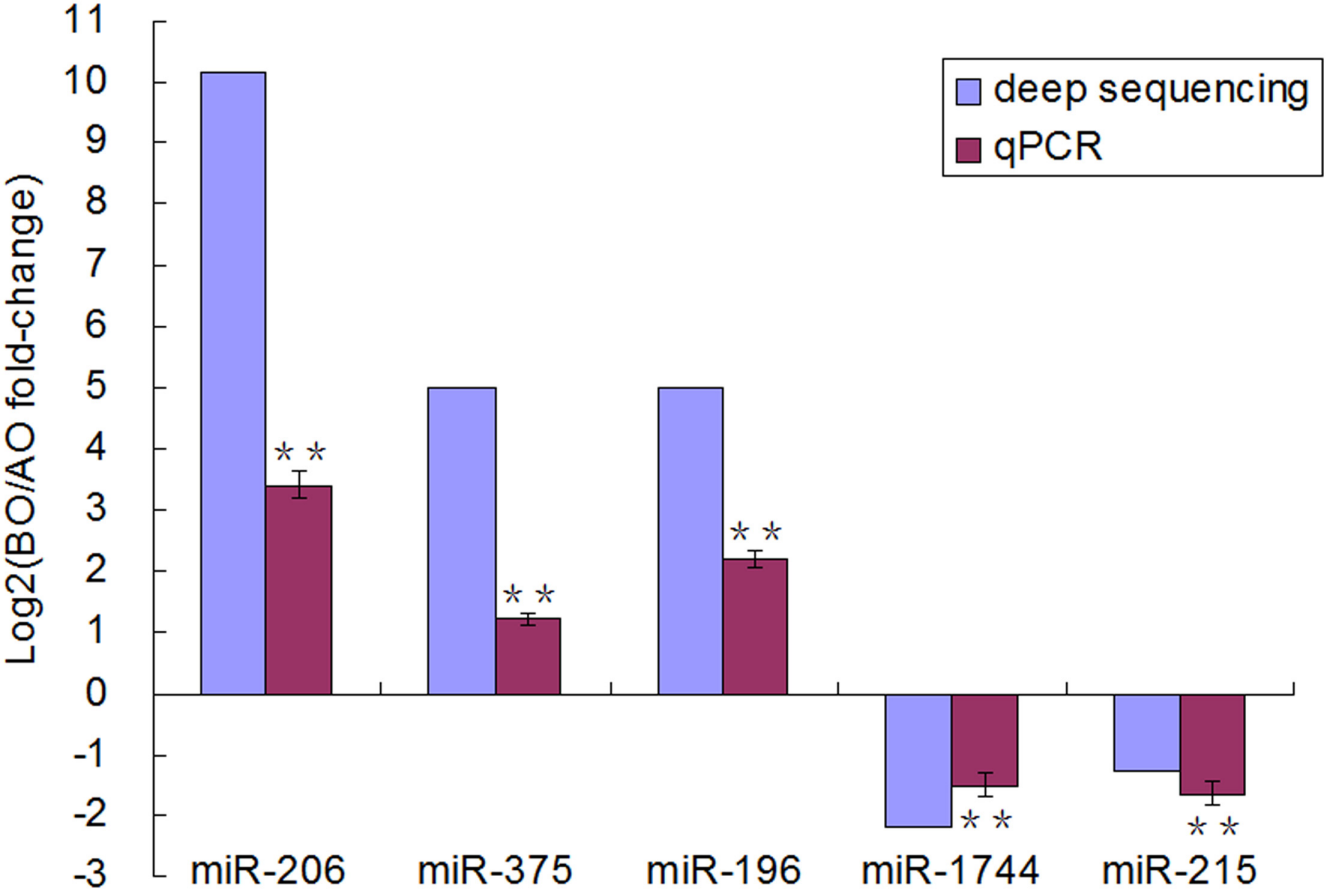
qRT- PCR validation of differentially expressed miRNAs supported by Solexa sequencing. Values shown are Log2(fold-change) in BO versus AO expression levels. qPCR results were normalized to U6 snRNA expression levels, error bars indicate standard errors for replicates (n = 3). The P values are calculated based on a t-test of the replicate values for each miRNA in BO and AO. **means a statistically significant difference (*P* < 0.01). BO, before onset of the rapid gonad development; AO, after onset of the rapid gonad development.

To validate the rapid gonad development onset related miRNAs detected by Solexa sequencing in chicken hypothalamus, qRT-PCR analysis of 5 differentially expressed miRNAs was performed. The primers sequences for validation of miRNAs were listed in S4 Table. As illustrated in Fig 2, these miRNAs showed similar expression trends with the results from Solexa sequencing. But the changed amplitudes in qRT-PCR were relatively smaller than that in Solexa sequencing.

### Target prediction and functional analysis of differential expression miRNAs

**Fig 3:**
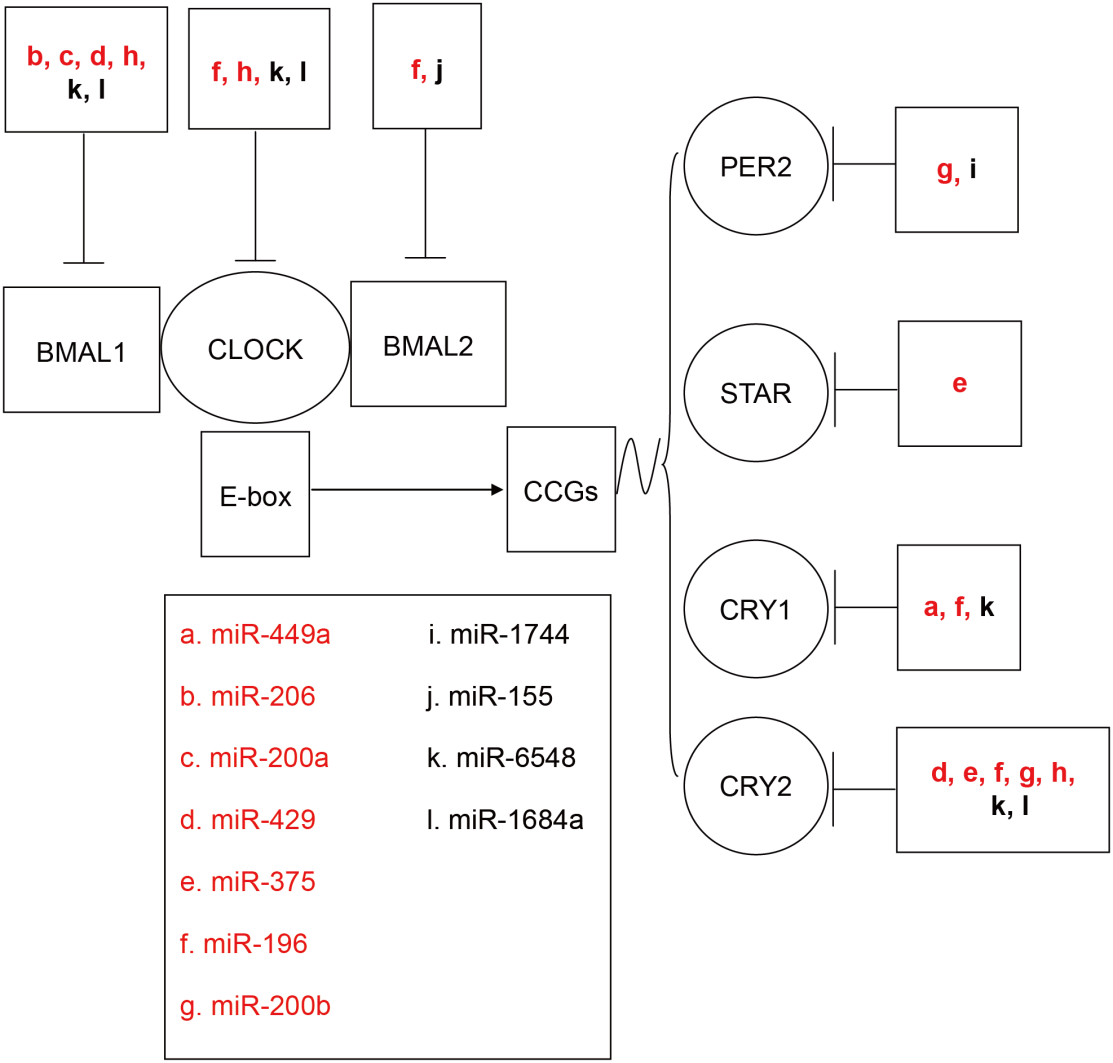
The circadian clock genes are predicted to be targeted by many differentially expressed miRNAs. Seven genes, *Per2*, *Bmal1/2*, *Clock*, *Cry1/2*, and *Star* implicated in circadian clock process, are predicted to be targeted multiple times by the differentially expressed miRNAs. In red: down-regulated (|log2(fold-change)|> 2.0, *P* < 0.01) miRNAs; in black: up-regulated (|log2(fold-change)|> 2.0, *P* < 0.01) miRNAs. BO, before onset of the rapid gonad development; AO, after onset of the rapid gonad development.

**Fig 4:**
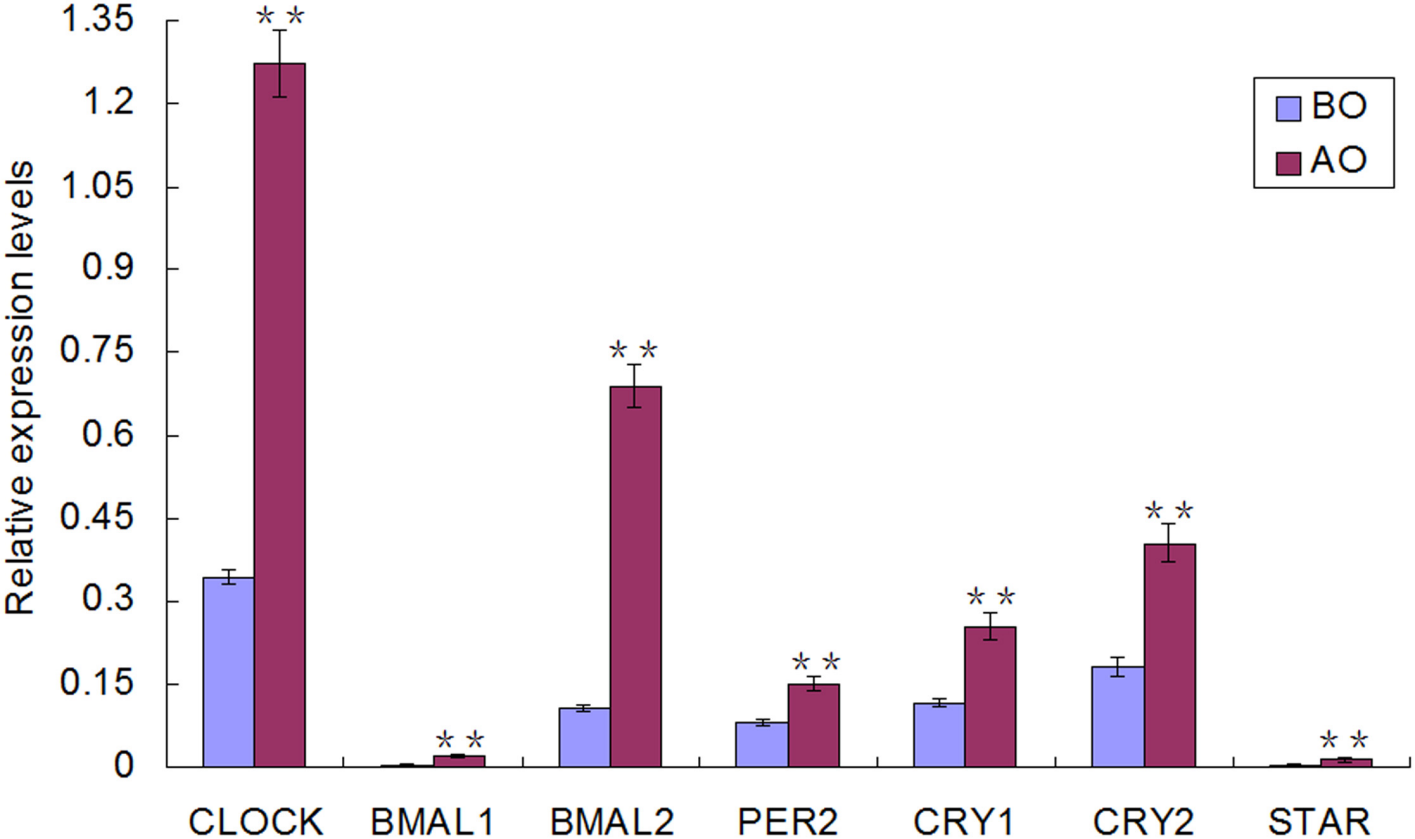
qRT- PCR validation of putative target genes. Mean expression levels were normalized to GAPDH controls, error bars show standard errors for replicates (n = 3). **means a statistically significant difference (*P* < 0.01). BO, before onset of the rapid gonad development; AO, after onset of the rapid gonad development.

To better understand the roles of differentially expressed miRNAs in controlling onset of the rapid gonad development, putative target genes of 6 up-regulated and 9 down-regulated miRNAs with |Log2 (fold-change)|> 2.0 were predicted by integrating TargetScan and miRanda software. In total, 2013 common target genes were found (data is not shown). Notably, seven circadian clock genes, *Per2*, *Bmal1/2*, *Clock*, *Cry1/2*, and *Star* were found to be targeted multiple times by these 15 miRNAs (Fig 3). qRT-PCR method was adopted to validate them. The primers sequences for validation of putative target genes were listed in S4 Table. The results (Fig 4) showed their expression levels all increased significantly (*P* < 0.01) from BO to AO.

GO analysis of the putative target genes (S5 Table) showed the putative target genes were significantly enriched (*P* < 0.05) in different groups. In molecular function (MF) level, the GO terms: Protein binding, ATP binding, Nucleotide binding, Zinc ion binding and DNA binding had significantly higher counts (counts > 100, *P* < 0.05). In biological process (BP) level, the GO terms: Regulation of transcription, DNA-dependent, Protein amino acid phosphorylation, Oxidation reduction and Intracellular protein transport had significantly higher counts (counts > 50, *P* < 0.05).

KEGG analysis of the putative target genes (S6 Table) suggested that MAPK signaling pathway, Ubiquitin mediated proteolysis, Cell cycle, Purine metabolism, Calcium signaling pathway and Wnt signaling pathway were among the most significant pathways (counts > 20, *P* < 0.01) related to chicken puberty onset.

## Discussion

To explore the regulatory mechanisms that control onset of the rapid gonad development, an essential prerequisite is to accurately measure the pubertal stages. In the last decade, there is no new measure emerging. Tanner method remains the primary system used for pubertal division in humans. The stage II, in which the thelarche and pubarche appear, indicates entry into puberty for girls. Vaginal opening (VO) is often taken as indicator of puberty onset in female mammals [23]. Measurement of reproductive hormone concentrations in serum or saliva [24, 25] has also been used to indicate pubertal stages. However, to date there is no systemic methodology can be used to measure developmental stages in the chicken. For hens, the external features can be observed during early development are changes of their crown sizes and colors, as well as body weights. But these are not precise indexes to time pubertal stages. In the present study, we roughly determined the timing of rapid gonad development onset in Wenchang hens by measuring the progressive development changes of gonad tissues. This provided a basis for constructing small RNA sequencing libraries that represented before and after onset of the rapid gonad development status respectively. Circulating LH concentration was also measured in our study, as with previously reported tendency, its level kept low in early period and did increase markedly at the 13 week. This LH increase coincided with the rapid growth of gonads. Then onset of the rapid gonad development in chickens might be thought of as analogous to puberty onset in mammals, which also correlated with increased LH pulsatility. However, it was difficult to discriminate whether an individual had entered into rapid growth of gonads based on LH, because the values showed wide deviations among individuals in one group. This phenomenon has also been observed in other studies [26]. So, in future it is necessary to find new type and more accurate markers, for example the circulating miRNAs [27], to measure pubertal stages.

It is evident that miRNAs are expressed in tissue-specific or stage-specific manner. The hypothalamus, as the key component of the central nervous system (CNS), is a major site of miRNA expression [28]. Several studies have detected large number of miRNAs are expressed in the hypothalamus of mouse [29–31], zebrafish [32] and chicken [33]. Hypothalamic miRNAs are showed to play roles in central regulation of energy homeostasis [34], circadian rhythms [35, 36], stress response, lactation and parturition [37]. With respect to puberty onset regulation by hypothalamic miRNAs, reports from Alvarellos *et al* (2013) [22] provide the most powerful evidence, extending beyond the model organisms to mammals. In rat hypothalamus, *Lin28*, *Lin28b* and *c-Myc* expression levels decrease dramatically during the infantile to juvenile transition and reach minimal levels before puberty. Conversely, let-7a, let-7b, miR-132 and miR-145 show opposite expression profiles. Changes in the *c-Myc*/*Lin28b*/let-7 pathway are also detected in models of delayed puberty by manipulation. Despite this considerable progress, the role of miRNAs in puberty onset is only beginning to be uncovered.

Given that high-throughput small RNA sequencing often yields large amounts of miRNAs, so pooling tissues from several individuals with complex backgrounds for sequencing would disturb discovery of the purposed miRNAs. In the present study, we set a cock family and collected hypothalamus from several full-/half-sib individuals with similar phenotypes to construct sequencing libraries. This experimental design may decrease the potential to find novel miRNAs, but can help us to identify related miRNAs. Here, we identified a total of 374 conserved miRNAs and 46 novel miRNAs in the chicken hypothalamus. Many of these miRNAs have been identified by others as the hypothalamus-expressed miRNAs. 5 randomly selected miRNAs were also validated by qRT-PCR. Thus, it suggested the Solexa sequencing method was reliable to identify and quantify hypothalamus-expressed miRNAs.

The 144 differentially expressed (*P* < 0.05) miRNAs between BO and AO were the focus of our attention. As expected, the let-7 family was contained in the list. Eight members were differentially expressed, but their relative abundance and expression changes were not consistent. Three members, let-7a, let-7b and let-7g expression levels increased, conversely, the other five members, let-7c, let-7f, let-7i, let-7j and let-7k expression levels decreased from BO to AO. It revealed the diverse functions of the let-7 family members. The increased expression trends for let-7a and let-7b have also been observed in hypothalamus of rat during puberty onset [22] and in whole brains of aging mice [38]. However, let-7a, let-7b and let-7g expression levels are showed to decrease in the hypothalamus of 1-day-old and 36-week-old chicken [33]. The reason for this difference may be the different examination ages.

Except for the let-7 family, totally 15 miRNAs were found to have more than four times expression changes (|log2fold-change| > 2.0) between BO and AO. Although the functions of these differentially expressed miRNAs in the hypothalamus are poorly understood, recent studies have suggested some are implicated in regulation of development and reproduction. miR-206 is showed to have profound effect on the timing of the circadian cycle in mammals [39]. miR-200b and miR-429 are linked to function in mouse ovulation and fertility [40]. miR-449 clusters are showed to be essential in brain development, motile ciliogenesis and spermatogenesis [41]. miR-375 is found to participate in the process of ovary maturation in chicken [42]. The physiological functions of miR-1744, miR-155, miR-6548, miR-1684a, miR-1665 and miR-6557 are still not known, but their significant expression changes from BO to AO indicate that they are likely to function in chicken rapid gonad development onset. Further experiments are needed to investigate their roles in this process. Intriguingly, most of these differentially expressed miRNAs have links with cancers. Previous studies have reported that many tumor suppress genes (TSG) are involved in the control of mammals or primates puberty onset [43, 44]. Whether these miRNAs fit into the TSG network needs to be explored.

Further evidence suggesting these differentially expressed miRNAs were relevant to onset of the rapid gonad development came from their predicted targets. Functional analysis showed that the putative 2013 target genes were involved in complex networks. Notably, among the putative target genes, seven circadian clock genes, including *Per2*, *Bmal1/2*, *Clock*, *Cry1/2* and *Star* were found to be targeted multiple times by the miRNAs. The intrinsic circadian clocks, consisting of transcriptional-translational feedback loop, have been found to reside in multiple components of the HPG axis in animals and humans [45, 46], and contribute to the timing of reproductive physiology, including estrous cycle, follicle maturation, pregnancy and parturition [47–49]. Considering the rhythmic patterns of reproductive hormones as well as metabolic factors release in pre-pubertal mammals [50–52] and humans [53, 54], the endogenous circadian clock may play a role in the initiation of puberty. Studies on genetic models have provided valuable cues. The *Bmal1* knockout female mice lose rhythmic secretion of reproductive hormones, have delayed puberty and smaller reproductive tissues [55, 56]. Although the mutation of *Clock* exerts slight influence on pubertal progression, adult female mice have lower fertility [57, 58]. In our study, the expression levels of the seven clock genes in the hypothalamus all increased significantly (*P* < 0.01) from BO to AO. Further, many of the clock genes are transcriptional factors and could alter the expression of genes involved in onset of rapid gonad development. Therefore, it is reasonable to deduce that the circadian genes and their partner miRNAs may be associated with onset of the rapid gonad development in chickens. In addition, prominent among the KEGG pathways, mammalian target of rapamycin (mTOR) signaling pathway has been found to couple the nutritional status to regulate puberty onset in rats [59] and the timing of molt in *Manduca sexta* [60]. ErbB signaling pathway has also been known to affect pulsatile release of GnRH, which is required for the timely initiation of puberty [61].

## Conclusions

The present study is the first to examine the miRNA expression profile in the hypothalamus of chickens during onset of the rapid gonad development. The results suggest that multiple miRNAs, not just restricted to the sole let-7 family, may be involved in timing the rapid development of chicken gonads. Due to highly conserved nature of miRNAs, the findings from our study will provide cues for human precocious puberty research.

## Supporting Information

S1 Table The conserved miRNAs expressed in the chicken hypothalamus. (XLS)

S2 Table Novel miRNAs expressed in the chicken hypothalamus. (XLS)

S3 Table The differentially expressed miRNAs between BO and AO. The miRNAs with |log2(fold-change)|> 2.0 were indicated in bold.
(XLS)

S4 Table The primer sequences for validation of miRNAs and putative target genes. (XLS)

S5 Table GO analysis of the putative target genes. (a) GO terms for cellular component; (b) GO terms for molecular function; (c) GO terms for biological process. Go terms with counts of genes > 50 were indicated in bold.
(XLS)

S6 Table KEGG analysis of the putative target genes. (XLS)
